# Impact of Hormonal Contraceptives on HPV Dynamics in Adolescent Girls and Young Women: Insights from a Randomized Controlled Sub-Study in South Africa

**DOI:** 10.1101/2025.01.14.25320519

**Published:** 2025-01-15

**Authors:** Ramla F. Tanko, Ongeziwe Taku, Zizipho Z. A. Mbulawa, Keletso Phohlo, Iyaloo Konstantinus, Christina Balle, Tanya Pidwell, Anna-Ursula Happel, Katherine Gill, Linda-Gail Bekker, Heather B. Jaspan, Anna-Lise Williamson, Jo-Ann S. Passmore

**Affiliations:** 1 Institute of Infectious Disease and Molecular Medicine (IDM), University of Cape Town, South Africa; 2 The Medical Research Centre, Institute of Medical Research and Medicinal Plant Studies (IMPM), Ministry of Scientific Research and Innovation, Yaoundé, Cameroon; 3 Department of Laboratory Medicine and Pathology, Walter Sisulu University, Mthatha, Eastern Cape, South Africa; 4 National Health Laboratory Service, Mthatha, South Africa; 5 Division of Immunology, University of Cape Town, South Africa; 6 Desmond Tutu Health Foundation, Cape Town, South Africa; 7 Seattle Children’s Research Institute, Seattle, USA; 8 Department of Science and Technology-National Research Foundation CAPRISA Centre of Excellence in HIV Prevention, Cape Town, South Africa; 9 National Health Laboratory Service, Cape Town, South Africa; 10 Namibia Institute of Pathology, Windhoek, Namibia

**Keywords:** Human papillomavirus, HPV, adolescents, persistence, contraceptives, Net-EN, COCPs, CCVR

## Abstract

**Objectives:**

Human papillomavirus (HPV) is the leading cause of cervical cancer, with adolescent girls and young women (AGYW) in sub-Saharan Africa carrying a disproportionately high burden of infection. Hormonal contraceptives may influence HPV acquisition, persistence, and clearance, but evidence remains inconclusive. This sub-study aimed to evaluate the impact of different hormonal contraceptives on HPV prevalence and genotype distribution in AGYW.

**Methods:**

Ninety-eight HIV-seronegative AGYW aged 15–19 years from South Africa were randomized to receive one of three hormonal contraceptive methods: norethisterone enanthate (Net-EN) injectable, combined oral contraceptive pills (COCPs), or the etonorgesterol/ethinyl estradiol combined contraceptive vaginal ring (CCVR). Cervical DNA samples were collected at baseline and after 16 weeks for HPV genotyping using the HPV Direct Flow Chip test. HPV prevalence, persistence, clearance, and acquisition were analyzed across contraceptive methods.

**Results:**

At baseline, HPV prevalence was high (94.9%), with no differences among contraceptive arms. After 16 weeks, HPV prevalence remained high (89.5%) across groups. No significant differences were observed in overall HPV prevalence or genotype distribution by contraceptive method. Longitudinal analysis revealed that AGYW using Net-EN tended to have a higher cumulative number of high-risk HPV (HR-HPV) genotypes that cleared whereas those using CCVR acquired more HR-HPV types and had greater HR-HPV persistence compared to other groups.

**Conclusions:**

This study highlights the high burden of HPV among South African AGYW. However, different hormonal contraceptive methods did not significantly influence HR-HPV dynamics.

## INTRODUCTION

Human papillomavirus (HPV) is one of the most prevalent sexually transmitted infections (STIs) affecting the human genital tract (1). Of the over 200 identified HPV types, only certain mucosal types are classified as high-risk (HR) due to their strong association with cancers (2,3). HPV-16 and HPV-18 are responsible for ~70% of cervical cancer cases globally and contribute significantly to other HPV-related malignancies, including anal, vaginal, vulvar, penile, and oropharyngeal cancers. Cervical cancer remains particularly widespread in sub-Saharan Africa, where incidence rates surpass those of other regions (4,5).

Risk of HPV infection is likely to be influenced by a combination of environmental, biological, and behavioral factors. Biologically, an impaired immune system, such as in individuals with HIV/AIDS, increases susceptibility to persistent HPV infections, while genetic predispositions (6) and co-infections with other STIs further elevate the risk (7). Key behavioral factors include early sexual debut, unprotected sex, multiple sexual partners, smoking, and prolonged use of hormonal contraceptives—all of which are associated with heightened risks of infection and cervical cancer (8). Understanding these complex factors is essential for developing targeted prevention and intervention strategies.

Observational studies have suggested that hormonal contraceptives may influence HPV dynamics (9–11). For instance, combined oral contraceptive pills (COCPs) have been associated with reduced HPV clearance, though other studies found no effect except with extended exposure. Meta-analyses suggest prolonged hormonal contraceptive use is linked to invasive cervical cancer (12,13), yet evidence on its relationship with HPV detection remains inconclusive.

Adolescent girls and young women (AGYW) in South Africa carry a high burden of HPV infection (14). Many of these AGYW are within their first year of sexual debut, with a median of one lifetime sexual partner (15), underscoring the alarming overlap between high HPV prevalence and vaccine coverage gaps.

This study investigated HPV prevalence and genotype distribution at baseline and after 16 weeks of hormonal contraceptive use in AGYW randomized to receive norethisterone enanthate (Net-EN), etonorgesterol/ethinyl estradiol combined contraceptive vaginal ring (CCVR), or COCPs. Importantly, data on HPV infection and CCVR use are limited. Understanding the impact of hormonal contraceptives on HPV dynamics is critical for guiding effective vaccination and reproductive health strategies.

## METHODS AND MATERIALS

### Description of study cohort

Between September 2015 and July 2017, 130 AGYW between the ages of 15 and 19 were recruited from the Desmond Tutu Health Foundation Youth Centre in Cape Town into an open-label, randomized crossover study evaluating the acceptability, feasibility, and adherence to contraceptive choices (uChoose study; clinicaltrials.gov number NCT02404038) (15). Eligible participants were HIV-seronegative, sexually active, not pregnant, had no symptomatic STIs that required antibiotic treatment in the 40 days prior to enrolment, and agreed not to use non-study vaginal products for the duration of the study. Following screening (baseline visit), eligible AGYW were randomized 1:1:1 to receive one of three hormonal contraceptives: NET-EN (200 mg), COCPs (Triphasil^®^ or Nordette^®^; both containing ethinyl estradiol and levonorgestrel), or CCVR (Nuvaring^®^; etonogestrel/ethinyl estradiol) for 16 weeks. AGYW who were <18 years provided written assent, and informed consent was obtained from their parents or legal guardians. AGYW who were ≥18 years old provided written informed consent. Ethical approval for the study was obtained from the University of Cape Town Human Research Ethics Committees (UCT HREC 801/2014).

### Testing for bacterial STIs and Bacterial vaginosis (BV)

At the time of sampling, vulvovaginal swabs were screened for *Chlamydia trachomatis, Neisseria gonorrhoea, Trichomonas vaginalis, and Mycoplasma genitalium* using a real-time multiplex PCR (16). BV was diagnosed using Nugent scoring, with Nugent Scores of 7–10 indicating a BV positive result (17). Vaginal pH levels were measured using color-fixed indicator strips (Macherey-Nagel, Düren, Germany). Participants who tested positive for STIs and/or BV received treatment according to the South African National guidelines.

### HPV genotyping

HPV genotyping was performed at baseline and after 16 weeks on extracted DNA (MagNA Pure Compact Nucleic Acid Isolation Kit; Roche), from endocervical swabs collected during the speculum examination, using the HPV Direct Flow Chip test (Master Diagnóstica, Granada, Spain), according to the manufacturers’ instructions. The Direct Flow Chip detects 36 different HPV genotypes, including the 12 HR types according to the International Agency for Research on Cancer (IARC) (3) (16, 18, 31, 33, 35, 39, 45, 51, 52, 56, 58, and 59), an additional 5 HPV types previously considered HR (26, 53, 66, 73, 82; according to the kit manufacturers), and 18 low-risk (LR) types (6, 11, 40, 42, 43, 44, 54, 55, 61, 62, 67, 69, 70, 71, 72, 81, 84, 89). For the purposes of HR-HPV analysis, the recent IARC (3) definition of HR-HPV types was used. For the purposes of LR-HPV analysis, the manufacturers definition of LR-HPV types was used. A sample was considered HPV positive when at least one HPV genotype was detected. Specimens testing positive for 2 or more HPV genotypes were considered to have multiple infections. Acquiring a new HPV infection was defined as detection of any HPV type at 16 weeks in AGYW who did not have that HPV type present at baseline. HPV persistence was defined as having the same HPV genotype detected at both baseline and 16 weeks, in the same participant. HPV clearance was defined as women testing negative for any HPV genotype at 16 weeks that was present at baseline.

### Statistical analyses

Fisher’s exact test and descriptive statistics were used to compare differences between hormonal contraceptive arms for categorical variables. Continuous variables were described using median and interquartile range (IQR) and compared using the Kruskal Wallis-test. P<0.05 was considered statistically significant. Statistical analyses were performed using GraphPad Prism version 9 (GraphPad Software, USA).

## RESULTS

Of the 130 AGYW enrolled into the parent uChoose study, endocervical samples were available for HPV genotyping from 98 women at baseline (74.5%) and 95 at 16 weeks (73.1%; Supplementary Figure 1). This included 36 matched sample pairs (baseline and 16 weeks) from adolescents randomized to receive Net-EN, 25 matched samples from AGYW randomized to receive COCPs, and 26 who were randomized to receive the CCVR.

**Figure 1.**
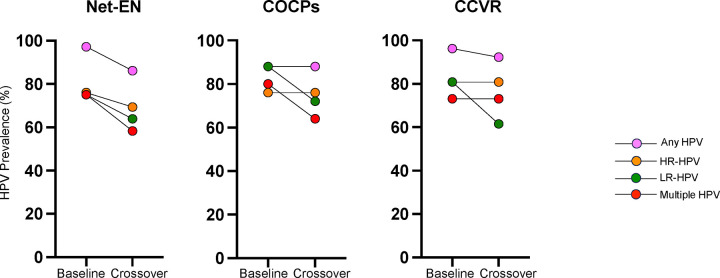
Dynamics of HPV Infections Across Contraceptive Methods. This figure illustrates the percentage of AGYW with cleared, persistent, and newly acquired HPV infections, based on paired data between baseline and week 16 visits. Results are stratified by hormonal contraceptive methods: CCVR (n=26), COCPs (n=25), and Net-EN (n=36). Color coding reflects infection categories: pink for any HPV, orange for HR HPV, darker blue for LR-HPV, and lighter blue for multiple HPVs.

**Figure 2.**
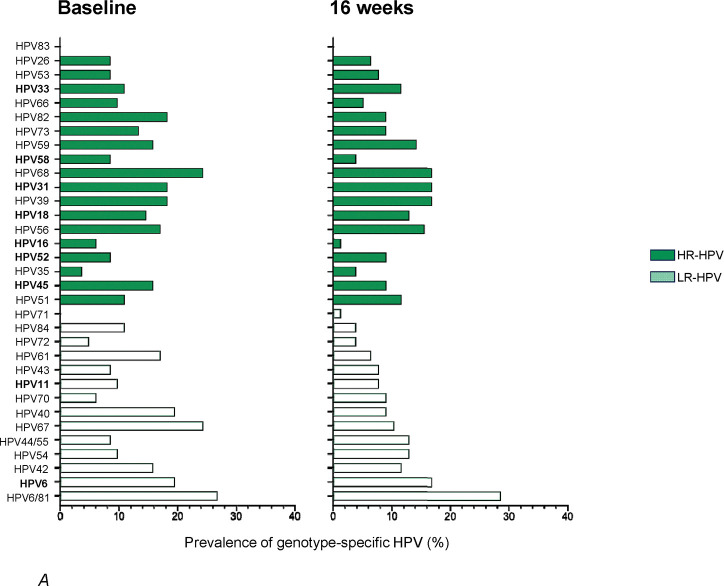

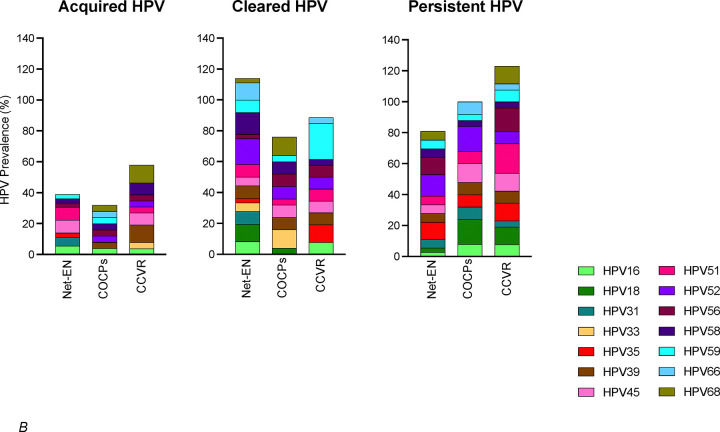
HPV Genotype Distribution Across Contraceptive Arms. (A) Distribution of HPV genotypes among AGYW at baseline and 16 weeks. Each bar graph represents the prevalence of a specific HPV genotype. Light colors indicate low-risk HPV (LR-HPV) types, while dark colors represent HR-HPV types. Genotypes no longer considered by IARC (3) to be HR are shown in green italics. Genotypes targeted by the bivalent (HPV 16, 18), quadrivalent (HPV 6, 11, 16, 18), and nonavalent (HPV 6, 11, 16, 18, 31, 33, 45, 52, 58) vaccines are highlighted in bold. (B) Stacked bars show the prevalence of HR HPV types (including HPV-66 and −68 which were previously classified as HR) that were newly acquired between 0 and 16 weeks (left panel), cleared (middle panel), or persisted (right panel), stratified by genotype and contraceptive method. Panels represent data for Net-EN (n=36), COCPs (n=25), and CCVR (n=26), based on paired samples between baseline and week 16 visits.

Demographics, genital infections, sexual risk behaviors, prior hormonal contraceptive use, and endogenous hormone levels did not significantly differ among hormonal contraceptive methods at baseline (Table 1). At baseline, HPV prevalence was high, with an overall prevalence of 94.9% (93/98), which did not differ between randomization arms (Figure 1 and Table 2). Most (80.5%) AGYW had multiple infections with HPV. Baseline prevalence of any HR-HPV was 78.9%. HPV-16 and HPV-18 were detected in 6/98 (6.1%) and 14/98 (14.2%) AGYW, respectively, with HPV-31 and HPV-39 being the most commonly detected HR type (both 18/98; 18.4%) (Figure 2A). While South Africa initiated the Cervarix (HPV-16/18) vaccination program a year before recruitment for uChoose began (19), which targeted grade 4 learners (aged 9 years or older), the AGYW enrolled in this study were unlikely to have been vaccinated as no catch-up vaccines were offered. The 9-valent Merck HPV vaccine would improve coverage although this does not contain other common HR-HPV types found in this cohort (HPV-39, HPV-56 [16/98; %], HPV-59 [14/98; 14.2%]; Figure 2A).

At 16 weeks, the HPV prevalence remained high (89.5%). Overall HPV prevalence and HR-HPV prevalence did not differ significantly by study arm (Figure 1 and Table 2). AGYW randomized to the Net-EN arm had an HPV prevalence (any) of 86.1% at 16 weeks compared to 97.2% at baseline (31/36 vs 35/36, p=0.2), those using COCPs had the same HPV prevalence at baseline and 16 weeks (both 88.0% [22/25]; p=1.0), and those randomized to the CCVR group had an HPV prevalence of 96.2% at 16 weeks compared to 92.3% at baseline (25/26 vs 24/26; p=1.0). For HR-HPV types, 66.7% of women randomized to Net-EN had a HR-HPV type at 16 weeks compared to 80.6% at baseline (24/36 vs 29/36, p=0.3), 56.0% of those using COCPs had HR-HPV infections at week 16 compared to 72.0% at baseline (14/25 vs 18/25; p=0.4), and those randomized to the CCVR group had an HPV prevalence of 76.9% at 16 weeks compared to 80.8% at baseline (20/26 vs 21/26; p=1.0).

Longitudinal analyses of HR-HPV genotype distribution showed no significant differences between the hormonal contraceptive arms, whether considering women who cleared infections (by type), had persistent infections (same type), or acquired new HPV genotype infections (by type; Figure 2B). However, there tended to be higher combined prevalence of new HR-HPV types acquired and that persisted in the CCVR arm compared to the other contraceptives arms. In contrast, adolescents in the Net-EN arm tended to have higher combined prevalence of HR-HPV counts cleared during the study than the other two arms.

## DISCUSSION

This sub-study of a randomized controlled trial (15) compared HPV prevalence and genotype distribution among AGYW randomized to three common hormonal contraceptives: CCVR, the injectable Net-EN, and COCPs. Our results revealed high HPV prevalence across all contraceptive methods at baseline, with no significant differences in overall prevalence or genotype distribution linked to any specific contraceptive arm after 16 weeks of use. Longitudinal analyses showed some variations in HR-HPV dynamics over the 16-week study period, although these were not significant. AGYW using Net-EN tended to clear more HR-HPV types, whereas those using the CCVR tended to acquire more HR-HPV infections that persisted compared to those in the other contraceptive arms.

The high HPV prevalence observed in this cohort aligns with other studies indicating that AGYW are at significant risk of acquiring HPV infections shortly after sexual debut (20,21). This rapid increase, often within the first six months of sexual activity, is influenced by factors such as sexual behaviors, the number of sexual partners, and immune response (22). In this study, an early average age of sexual debut (13 years) and additional risk factors, such as inconsistent condom use (43.9%), multiple sexual partners (21.4%), and high rates of other STIs (e.g., *C. trachomatis* prevalence of 31.6%), likely contributed to the high HPV rates at baseline.

We have previously shown that hormonal contraceptives exert distinct effects on the mucosal environment of the female genital tract, as evidenced by changes in microbial composition (23), inflammatory markers (24), and gene expression (25). In this cohort, adolescents randomized to Net-EN exhibited higher vaginal microbial diversity and proinflammatory taxa compared to those using COCPs, who had associated with increased lactobacilli and reduced genital inflammation (23,24). CCVR use, however, was linked to elevated transcriptional networks involving interleukin (IL)-6, IL-1, and NF-κB, suggesting increased genital tract inflammation, alongside reduced epithelial barrier integrity (25). As altered vaginal microbiota (26), reduced genital barrier integrity (27) and chronic inflammation (28) have all previously been associated with increased risk for HPV infection and carcinogenesis, this may explain our observation that AGYW using the CCVR might be more likely to acquire and retain HR-HPV infections compared to other contraceptive users.

The findings suggest that hormonal contraceptives may differentially impact HR-HPV acquisition, persistence, and clearance. The observed benefits of Net-EN in clearing HR-HPV types and the increased acquisition and persistence associated with CCVR underscore the need to explore biological mechanisms underlying these effects. These insights are critical for tailoring contraceptive counseling and HPV prevention strategies, particularly in high-risk populations like AGYW in sub-Saharan Africa. Further research is essential to validate these findings and assess the long-term implications for cervical health and cancer prevention.

## Supplementary Material

Supplement 1

## Figures and Tables

**Table 1.** Baseline characteristics of AGYW in UChoose HPV sub-study

Contraceptive arm	All	Net-EN^a^	COCP	CCVR^b^
n	98/130 (75.4%)	44/98 (44.9%)	24/98 (24.5%)	25/98 (25.5%)
Age (years)*	17.0 (16.0 – 18.0)	17.0 (16.0 – 18.0)	16.0 (16.0 – 18.0)	17.0 (16.0 – 18.5)
Body mass index (kg/m^2^)*	25.1 (21.5 – 29.1)	25.8 (22.6 – 29.8)	25.5 (20.7 – 30.5)	24.9 (21.1 – 26.5)
Age of sexual debut (years)*	13 (12.0 – 14.0)	13 (12.0 – 14.0)	14 (12.0 – 15.0)	13 (12.0 – 15.0)
Condom use during last sex	55/98 (56.1%)	24/44 (54.5%)	14/24 (58.3%)	15/25 (60.0%)
>1 sex partners in past year	21/98 (21.4%)	10 (27.8%)	5 (20.8%)	6 (24.0%)
New partners in past year	28/98 (28.6%)	10 (22.7%)	7 (29.2%)	10 (40.0%)
Prior hormonal contraceptive use:				
Never	1/98 (1.0%)	1 (2.3%)	0 (0.0%)	0 (0.0%)
Not currently	21/98 (21.4%)	9 (20.5%)	7 (29.2%)	4 (16.0%)
DMPA	9/98 (9.2%)	4 (9.1%)	4 (16.7%)	1 (4.0%)
Implanon	2/98 (2.0%)	1(2.3%)	0 (0.0%)	1 (4.0%)
COCPs	3/98 (3.1%)	2 (4.5%)	0 (0.0%)	1 (4.0%)
Net-EN	62/98 (63.3%)	27 (61.4%)	13 (54.2%)	18 (72.0%)
Vaginal pH*	4.9 (4.4 – 5.3)	5.0 (4.7 – 5.3)	4.7 (4.4 – 5.0)	4.7 (4.4 – 5.3)
Bacterial vaginosis:				
Negative (Nugent 0 – 3)	46/98 (46.9%)	18/44 (40.9%)	12/24 (50.0%)	13/25 (52.0%)
Intermediate (Nugent 4 – 6)	10/98 (10.2%)	6/44 (13.6%)	1/24 (4.2%)	2/25 (8.0%)
Positive (Nugent 7 – 10)	42/98 (42.9%)	20/44 (45.5%)	10/24 (41.7%)	11/25 (44.0%)
STIs:				
*Chlamydia trachomatis*	31/98 (31.6%)	12/44 (27.3%)	9/24 (37.5%)	9/25 (36.0%)
*Neisseria gonorrhoeae*	12/98 (12.2%)	5/44 (11.4%)	3/24 (12.5%)	3/25 (12.0%)
*Trachomatis vaginalis*	8/98 (8.2%)	5/44 (11.4%)	1/24 (4.2%)	2/25 (8.0%)
*Mycoplasma genitalium*	3/98 (3.1%)	2/44 (4.6%)	1/24 (4.2%)	0/25 (0.0%)

* Median and 25^th^ and 75^th^ percentile; n: sample size: sample size

a No record available for age of sexual debut (n=3), condom use during last sex (n=8), numbers of sexual partners (n=8), new partners (n=9) and endogenous hormone levels (n=2) and n=1

b No data available for endogenous hormone levels (n=1)

**Table 2.** HPV prevalence at baseline and 16 week visits for paired samples

HPV types	Baseline			16 weeks		
	Net-EN	COCP	CCVR	Net-EN	COCP	CCVR

Any HPV	35/36 (97.2%)	22/25 (88.0%)	25/26 (96.2%)	31/36 (86.1%)	22/25 (88.0%)	24/26 (92.3%)
HR-HPV	29/36 (80.60%)	18/25 (72.0%)	21/26 (80.8%)	24/36 (66.7%)	14/25 (56.0%)	20/26 (76.9%)
LR-HPV	27/36 (75.0%)	22/25 (88.0%)	21/26 (80.8%)	23/36 (63.9%)	18/25 (72.0%)	16/26 (61.5%)
Multiple HPV	27/36 (75.0%)	20/25 (80.0%)	19/26 (73.1%)	21/36 (58.3%)	16/25 (64.0%)	19/26 (73.1%)
