# Supplementary material for: Impact of Hormonal Contraceptives on HPV Dynamics in Adolescent Girls and Young Women: Insights from a Randomized Controlled Sub-Study in South Africa
Source: medRxiv. 2025 Jan 15:2025.01.14.25320519. Preprint. [Version 1] doi: 10.1101/2025.01.14.25320519 (PMC11759828; doi:10.1101/2025.01.14.25320519)
Supplement: Supplement 1 [file NIHPP2025.01.14.25320519v1-supplement-1.pdf]

medRxiv preprint doi: https://doi.org/10.1101/2025.01.14.25320519; this version posted January 15, 2025. The copyright holder for this preprint (which was not certified by peer review) is the author/funder, who has granted medRxiv a license to display the preprint in perpetuity.
This article is a US Government work. It is not subject to copyright under 17 USC 105 and is also made available for use under a CC0 license.

Supplemental Figure 1. Study design overview

Study design for the open-label, randomized crossover trial conducted among adolescent girls and young women (AGYW). Participants were randomized to one of three hormonal contraceptive methods: combined oral contraceptive pills (COCPs), injectable Net-EN, or combined contraceptive vaginal ring (CCVR). Each participant used their assigned contraceptive method for 16 weeks before crossing over to another method, with no washout period between interventions. The primary

medRxiv preprint doi: https://doi.org/10.1101/2025.01.14.25320519; this version posted January 15, 2025. The copyright holder for this preprint (which was not certified by peer review) is the author/funder, who has granted medRxiv a license to display the preprint in perpetuity. This article is a US Government work. It is not subject to copyright under 17 USC 105 and is also made available for use under a CC0 license.

outcomes were acceptability, feasibility, and adherence. Assessments were conducted at baseline, and 16 weeks. The crossover design allowed each participant to serve as their own control, enabling robust comparisons between the different contraceptive methods.

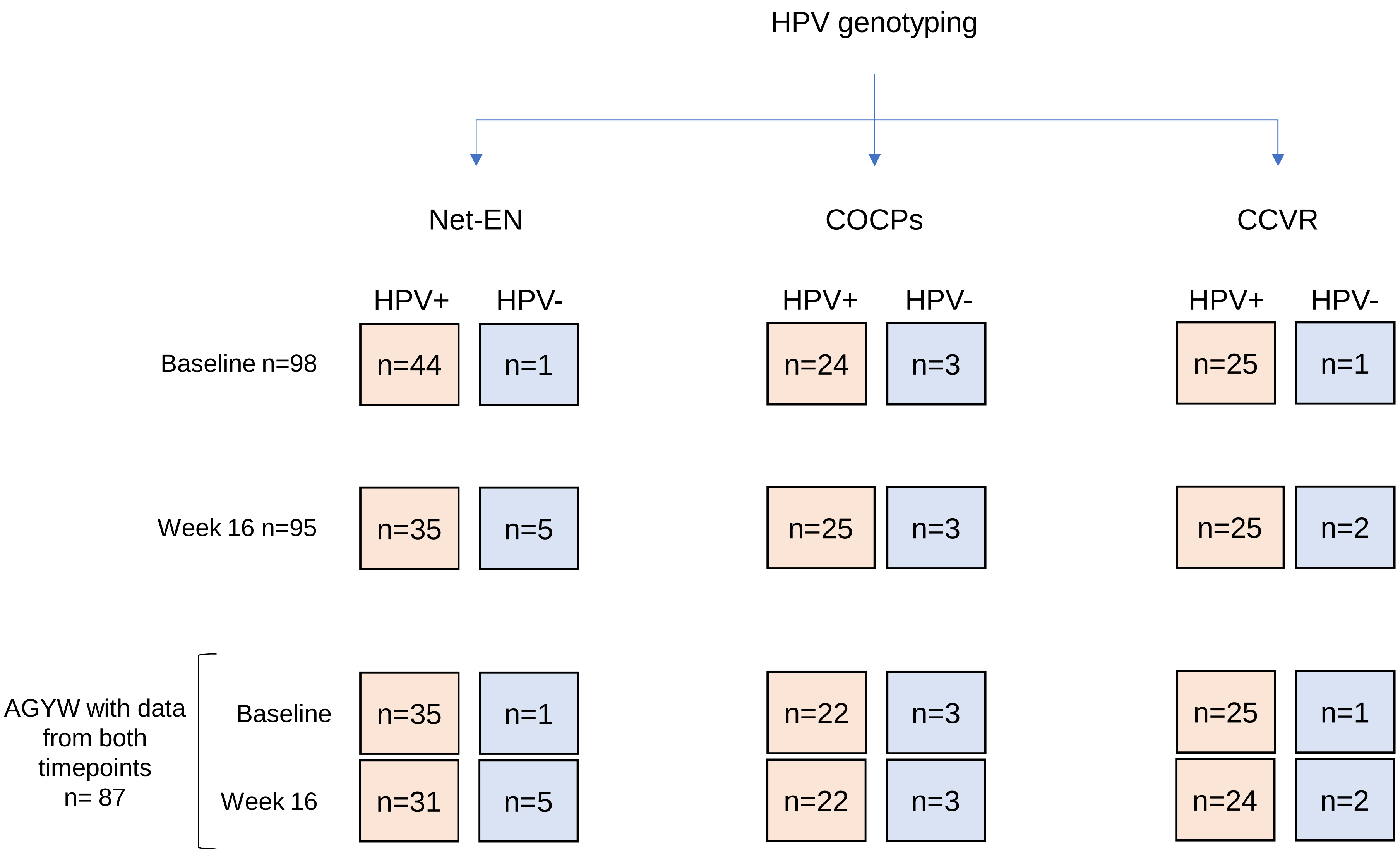

Supplemental Figure 1

medRxiv preprint doi: https://doi.org/10.1101/2025.01.14.25320519; this version posted January 15, 2025. The copyright holder for this preprint (which was not certified by peer review) is the author/funder, who has granted medRxiv a license to display the preprint in perpetuity. This article is a US Government work. It is not subject to copyright under 17 USC 105 and is also made available for use under a CC0 license.
